# Asprosin in pregnancy and childhood

**DOI:** 10.1186/s40348-020-00110-8

**Published:** 2020-12-23

**Authors:** Ruth Janoschek, Thorben Hoffmann, Yousef Ashraf Tawfik Morcos, Gerhard Sengle, Jörg Dötsch, Eva Hucklenbruch-Rother

**Affiliations:** 1 Department of Pediatrics, and Adolescent Medicine, Faculty of Medicine and University Hospital of Cologne, University of Cologne, Cologne, Germany; 2 Center for Biochemistry, Faculty of Medicine and University Hospital Cologne, University of Cologne, Cologne, Germany; 3 Center for Molecular Medicine Cologne (CMMC), University of Cologne, Cologne, Germany; 4 Cologne Center for Musculoskeletal Biomechanics (CCMB), Cologne, Germany

## Introduction

The prevalence for childhood overweight and obesity increased steadily in the past decades. Childhood obesity was defined as a disease by the World Health Organization [25] and by the American Medical Association (2013) [7] and is listed in the International Statistical Classification of Diseases and Related Health Problems in (ICD). In 2016 over 340 million children and adolescents, aged 5–19 were overweight or obese and current estimates suggest approximately 38 million children under the age of 5 as overweight or obese [25]. Amongst genetic factors and environmental influences, a variety of perinatal risk factors individually or in combination contributes to the development of infant obesity. Especially maternal pre-pregnancy obesity, excessive gestational weight gain and gestational diabetes result in pathological pregnancy conditions. Insulin resistance (IR), elevated blood glucose levels and hormonal disturbance provoke the malprogramming of infant’s energy metabolism [11].

**Fig. 1:**
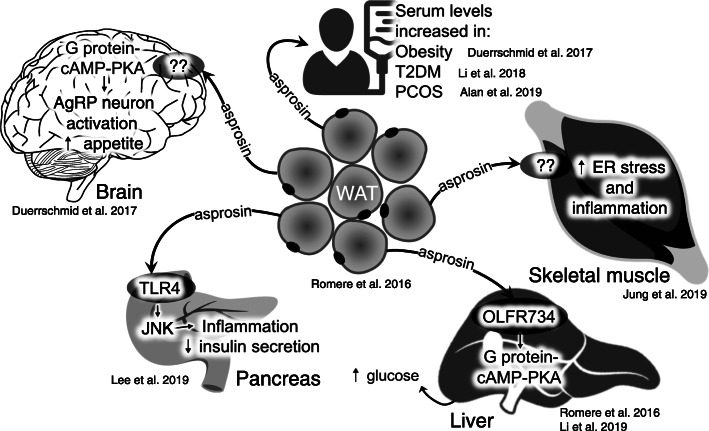
Overview of asprosin action in health and disease modified according to Muthu and Reinhardt [18]

In 2016, Romere et al. discovered asprosin as a new adipokine exerting decisive metabolic functions. Asprosin is the C-terminal peptide of the extracellular matrix protein fibrillin-1 that is cleaved off by the propeptide convertase furin during the secretory pathway [17]. According to the current paradigm, asprosin is released from white adipose tissue (WAT) into the blood stream to target metabolically relevant organs. Following a circadian oscillation, asprosin triggers hepatic glucose release via the OLFR734 receptor [14, 19] and impairs insulin secretion from pancreatic beta-cells [13]. Furthermore, asprosin crosses the blood–brain barrier, activates hunger-stimulating AgRP (Agouti-related peptide) hypothalamic neurons thereby evoking appetite stimulation in mice [6]. Also, insulin sensitivity in muscle cells is impaired by asprosin via the PKCδ/SERCA-2 pathway by increasing inflammation and ER stress [12]. Serum asprosin levels are elevated in obese humans and mice, and neutralisation of asprosin via antibody treatment reduces food intake and body weight as well as improves insulin sensitivity in mice [6]. Further studies revealed that asprosin levels were significantly higher in patients with impaired glucose regulation and correlated with a variety of clinical parameters of glucose and lipid metabolic disorders [24]. Furthermore, asprosin levels were significantly higher in women with type 2 diabetes mellitus (T2DM), and positively correlated with IR in women with polycystic ovary syndrome (PCOS) [1, 15]. These observations suggest asprosin as a potential biomarker of early diagnosis in metabolic-related diseases and a new therapeutic target not only for T2DM and other metabolic disorders but also for counteracting the perinatal programming of childhood obesity (overview see Fig. 1).

Currently, only little data regarding asprosin levels in pregnant women or children are available. So far, two clinical studies addressed maternal and newborn asprosin levels [3, 26] whereas three studies examined serum asprosin in overweight and obese children [16, 21, 23].

Zhong et al. examined 80 pregnant, non-obese women; 40 with gestational diabetes mellitus (GDM), and 40 with normal glucose tolerance (NGT), aged 18–40. All participants were without pre-existing diabetes mellitus (DM), history of macrosomia, nor stillbirth, polycystic ovary syndrome, nor medications of corticosteroids or antipsychotics. The authors performed asprosin measurements at three different time points, 18–20 gestational weeks (gws), 24–28 gws, as well as before delivery. Neonate asprosin protein concentrations were determined in umbilical cord plasma by ELISA, and additional asprosin protein expression analysis via western blots and immunohistochemistry were performed in placenta. Asprosin levels were elevated in plasma of pregnant women with GDM (18–20 gws and before delivery) and in newborns from GDM mothers. Asprosin levels correlated positively with maternal age but not with maternal BMI (18–20 gws and 24–28 gws). However, asprosin levels correlated negatively before delivery with maternal age and BMI. Neonatal asprosin levels showed a positive correlation with birthweight. The authors detected asprosin in the placenta but without any difference between GDM and NGT. Finally, they concluded that the “insulin resistance-related factor asprosin is expressed in placenta and is elevated in pregnant women complicated with GDM both before and after diagnosis, making it a potential predictor of GDM. Further research on the regulation of asprosin in placenta may improve the understanding of placenta induced insulin resistance mechanism [26].

Baykus et al. examined 30 pregnant women with GDM, preeclampsia (PE), severe preeclampsia (SPE) and intra-uterine growth restriction (IUGR) respectively; 29 pregnant women with macrosomic foetus (MF); and 30 healthy pregnant women at 37–39 gws. The age range of the participating women was 18–35, and the cohort had normal pregnancies without diabetes mellitus, any chronic disease and any form of medication, alcohol, or the use of tobacco products. Unfortunately, the presented data does not specify whether the pregravid or prepartum BMI (29–32 kg/m^2^) is reported. Furthermore, the authors took blood samples from umbilical cord (artery and vein) of newborns, and unfortunately, details on how the maternal blood draw was conducted are missing too. They state a positive correlation of maternal and newborn asprosin levels. Peculiarly, the authors display identical figures for maternal asprosin and venous umbilical cord asprosin levels. In GDM, PE, SPE and MF mothers and in their newborns, asprosin levels were significantly higher compared to the control group whilest maternal and offspring IUGR asprosin levels were significantly decreased. In all groups, female neonates displayed significantly higher asprosin levels than males. Baykus et al. concluded that their study “might help to enlighten the resolution of the mystery under gestational diabetes, IUGR, macrosomic foetuses, preeclampsia and severe preeclampsia etiology” [3].

Amongst studies addressing overweight and obese children, [16] analysed plasma samples of 47 obese children and 40 age-matched healthy controls, at 6–14 years of age, without family history of diabetes or other diseases, nor medical treatment regarding weight control. There were no significant differences in age and sex distribution, BMI, HOMA-IR and other markers between the two groups. Pubertal development was evaluated (Tanner stage). The detected asprosin levels were significantly lower in obese compared to normal-weight children (9.24 ± 4.11 vs 12.333 ± 4.18 ng/ml). Additionally, asprosin levels in obese boys were lower compared to girls of the same group. There were no sex differences in asprosin levels in the control group. Due to these findings, Long et al. concluded “a complex role for asprosin in energy metabolism” [16].

The second study in 2019 by Wang et al. examined 119 children with an average age of 10, 79 obese, 40 non-obese, 43 girls, 76 boys, all without taking any medication. There were no significant differences in terms of age, gender, and pubertal stage (Tanner); however, detailed specification according to age and gender are missing. Asprosin levels were significantly higher in obese compared to non-obese children but no differences between children according gender and Tanner stages could be observed. Further differentiation between mild to moderate obesity and severe obesity according to SDS-BMI was performed. Unfortunately, differences in asprosin levels were mostly but incompletely illustrated via dot-blots without determining total amounts. Results for asprosin levels in obese and non-obese subjects are just reported in the text. Detailed information (e.g. a table) presenting the measured asprosin concentration values are missing. Additionally, the authors performed several correlation tests and reported a positive correlation of circulating asprosin levels with IR. Finally, Wang et al. concluded that “Asprosin plays a pathogenetic role in insulin resistance […] asprosin is involved in the onset of obesity and may be a new biomarker of insulin resistance […] asprosin might serve as a risk factor associated with the development of diabetes mellitus type 2. Further studies are required to investigate whether asprosin could be a novel therapeutic target for obesity-related disorders”.

In 2020, a third study objecting asprosin in overweight and obese children was published by Sünnetçi Silistre and Hatipoğlu. Three groups of children (54 overweight, 44 obese and 60 normal-weight), composed of 77 girls and 81 boys, were examined. Unfortunately, the reported data are only partially comprehensible, as some of the displayed values remain unexplained. Asprosin levels were detected in fasted plasma. The authors report increased concentrations of asprosin in overweight and obese children compared to normal-weight children, whereby obese children displayed higher asprosin levels compared to overweight children. Overall girls had higher asprosin levels than boys. The study did not detect any other significant correlations between asprosin and additional metabolic factors. The authors report asprosin as a marker for childhood obesity and conclude “Further studies are needed to demonstrate the role of asprosin in the etiology of childhood obesity, as well as other diseases that might be associated with effects of asprosin” [21].

Overall, there are considerable differences between the individual study designs, which do not allow inter-study comparison nor gaining reliable basic knowledge of asprosin in pregnant women, newborns and older children. For instance, not only the inclusion criteria of study cohorts differ, but also the fasting status of individuals at the time point of sample collection: maternal and neonate’s asprosin was obviously detected in the fed state, either in plasma (Zhong) or in serum (Baykus), whereas children’s asprosin levels were measured in fasted serum (Wang) or fasted plasma (Long, Sünnetçi Silistre).

Despite these problems, the following major findings can be summarised:

1. Asprosin levels are elevated in GDM, both in mothers and neonates [3, 26].
2. Asprosin is expressed in the placenta [26].
3. Maternal asprosin levels correlate with newborn asprosin [3, 26].
4. Girls have higher asprosin levels than boys, both in newborns and in children [3, 16, 21].

However, a variety of ambiguities and open questions remain. The most serious concern is the determination of asprosin levels since the reported results show a high variability. For instance, asprosin blood concentrations of children with similar BMI vary between the five studies from < 1 ng/ml up to > 100 ng/ml (Table 1). Possible reasons for these discrepancies might be the quality of the individual detection kits. Although each workgroup lists an individual supplier, closer examination revealed that the ELISA kits used by Zhong et al. and Wang et al. (distributed by Abexxa and USCN Life Science) list the exact same components and assay range (0.156–10 ng/ml). In addition, both kits have a very similar sensitivity (< 0.057 ng/ml vs. 0.06 ng/ml) and follow a very similar protocol, suggesting that they are derived from the same original manufacturer. Both groups report comparable asprosin levels in pregnant women (Zhong) and children (Wang). However, their results contrast the asprosin concentrations measured by Long et al. (EIAab). Even though the set of ELISA kit components resembles the kits from Abexxa and USCN, the detection range of the EIAab kit is one order of magnitude higher (1.56–100 ng/ml) which may explain the approximately 10-fold higher asprosin measurements by Long et al. The kit used by Silistre et al. (Sinogeneclon), who report the highest amounts of asprosin (70–106 ng/ml), not only displays the highest detection range (7.8–500 ng/ml), but also clearly differs in sensitivity (2.1 ng/ml) and kit components. Finally, the kit used by Baykus et al. (Sunred Bioscience) is not listed anymore on the manufacturer’s site, so more detailed information on its components and characteristics could not be retrieved.

**Table 1:**
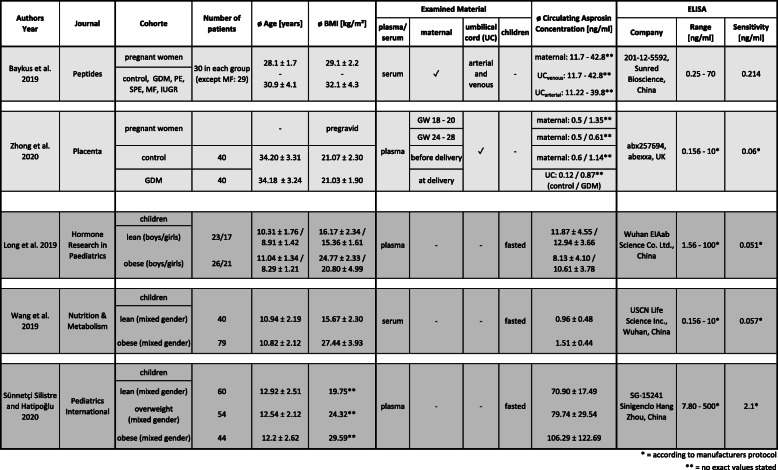
Comprehensive summary of existing asprosin studies in pregnant women and children

Consequently, a reliable determination of serum asprosin levels appears to be not yet feasible, thereby making a precise correlation of asprosin with obesity-related markers and other factors impossible. Therefore, the availability of standardised methods for asprosin analysis is mandatory. For example, the development of a universally applicable and sensitive asprosin ELISA seems to be a crucial requirement for reliable asprosin measurements in clinical samples. Thereby, it may be important to compare asprosin levels in different body fluids.

Typically, serum, plasma or urine are the most common biological matrices for the analysis of hormones, but the analysis of saliva seems to be a useful alternative especially in pediatrics as the collection of saliva is simple, non-invasive and painless [5, 10]. In 2019, Ugur et al. demonstrated a correlation of saliva with serum asprosin [22]; however, a recent publication observed different results comparing saliva and plasma [9]. Also, Bielohuby et al. reported significant differences in the concentrations of the metabolic hormones leptin and glucagon-like peptide-1 (GLP-1) when comparing serum and plasma [4] raising the question whether serum and plasma asprosin levels do correlate.

In the context of perinatal programming, also breast milk might be a promising subject of investigation. Metabolic hormones like leptin, ghrelin, insulin and GLP-1 are secreted into human milk. The amounts of some of these hormones are associated with the maternal BMI [2]. For example, leptin levels are higher in milk from overweight and obese mothers than normal weight mothers [8] and foremilk GLP-1 was correlated with maternal body weight whereas hindmilk GLP-1 was correlated with infant weight gain from birth to 6 months of age [20]. Consequently, it is likely that asprosin is secreted to human milk in proportion to maternal BMI and that it exerts metabolic effects on neonate organs.

In conclusion, further investigations of asprosin levels and asprosin correlations in various body fluids of mothers and children are required to get more insight into asprosin’s action and significance in pregnancy and childhood.

## Data Availability

Not applicable.

## Abbreviations

AgRP: Agouti-related peptide
BMI: Body mass index
DM: Diabetes mellitus
GDM: Gestational diabetes mellitus
GLP-1: Glucagon-like peptide-1
gws: Gestational weeks
HOMA-IR: Homeostasis model assessment for insulin resistance
ICD: International Statistical Classification of Diseases
IR: Insulin resistance
IUGR: Intrauterine growth restriction
MF: Macrosomic foetus
NGT: Normal glucose tolerance
PCOS: Polycystic ovary syndrome
PE: Preeclampsia
SPE: Severe preeclampsia
T2DM: Type 2 diabetes mellitus
UC: Umbilical cord
WAT: White adipose tissue
WHO: World Health Organization
